# Cloud service checklist for academic communities and customization for genome medical research

**DOI:** 10.1038/s41439-022-00214-9

**Published:** 2022-10-17

**Authors:** Kumiko Kobayashi, Hiroshi Yoshida, Tomoya Tanjo, Kento Aida

**Affiliations:** 1 National Institute of Informatics, Chiyoda-ku, Japan; 2 National Institute of Informatics, National Institute of Genetics, Mishima City, Japan

**Keywords:** Computational biology and bioinformatics, Medical research

## Abstract

In this paper, we present a cloud service checklist designed to help IT administrators or researchers in academic organizations select the most suitable cloud services. This checklist, which comprises items that we believe IT administrators or researchers in academic organizations should consider when they adopt cloud services, comprehensively covers the issues related to a variety of cloud services, including security, functionality, performance, and law. In response to the increasing demands for storage and computing resources in genome medical science communities, various guidelines for using resources operated by external organizations, such as cloud services, have been published by different academic funding agencies and the Japanese government. However, it is sometimes difficult to identify the checklist items that satisfy the genome medical science community’s guidelines, and some of these requirements are not included in the existing checklists. This issue provided our motivation for creating a cloud service checklist customized for genome medical research communities. The resulting customized checklist is designed to help researchers easily find information about the cloud services that satisfy the guidelines in genome medical science communities. Additionally, we explore whether many cloud service providers satisfy the requirements or checklist items in the cloud service checklist for genome medical research by evaluating their survey responses.

## Data storage: Cloud services checklist for medical genomics researchers

Criteria that will help researchers and IT administrators choose cloud service providers appropriate for the medical genomics research field have been formulated. Researchers collect and analyze increasingly large amounts of data, making cloud services essential. Although some guidelines have been published, security concerns make many researchers who handle sensitive data hesitant to adopt cloud services. Kumiko Kobayashi at the National Institute of Informatics in Tokyo, Japan, and co-workers have published a cloud services checklist customized for genome medical researchers that addresses concerns of security, functionality, performance, and law. Issues such as security (physical and encryption), disaster recovery, data deletion assurance, and laws governing disputes (for cloud services companies in different countries) are covered. This checklist should help researchers choose appropriate cloud services and improve access to big data services.

## Introduction

Cloud services are now used for many purposes, including research, education, and organization management, in a wide variety of academic communities. For example, a recent survey on academic information infrastructure conducted by the Ministry of Education, Culture, Sports, Science and Technology (MEXT) in Japan indicates that more than 90% of universities now use cloud services in their information systems^1^. In our current era, major data science communities, such as those pursuing life science fields, require computing systems that can archive and analyze large-scale scientific data. As a result, cloud services are rapidly becoming essential in new computing systems as well as conventional on-premise computing systems. For example, the National Institute of Genetics (NIG) in Japan has already begun utilizing a public cloud computing service in a hybrid collaboration with their on-premise supercomputer^2^, while the National Institutes of Health (NIH) in the United States has promoted the usage of public clouds in academic research through its Science and Technology Research Infrastructure for Discovery, Experimentation, and Sustainability (STRIDES) Initiative^3^.

However, security issues are matters of serious concern among information technology (IT) administrators in academic organizations. Indeed, the abovementioned MEXT survey results indicate that while higher security is the reason cloud services are used in 50% of universities that have adopted such services, it is also the reason 50% of universities have declined opportunities to adopt cloud services^1^. These contradictory survey results indicate that any standard criteria for the use of cloud services have not been adopted in Japanese academic communities and that this lack makes researchers hesitant to use cloud services—particularly in academic fields involving sensitive data, such as genome medical research. As a result, these researchers remain ignorant of opportunities to utilize the data and computing capacity offered by cloud services.

As criteria for the use of cloud services in genome medical science communities, guidelines for using the resources operated by external organizations, such as cloud services, have been published by different academic funding agencies. For example, the National Bioscience Database Center (NBDC) of the Japan Science and Technology Agency (JST) has published the “NBDC Guidelines for Human Data Sharing”^4^ (hereafter, “NBDC guidelines”), to define the rules for storing and analyzing human-related data generated using public funds while considering the protection of personal information. The NBDC guidelines include requirements for the operation of databases/servers operated by academic organizations outside of the NBDC. Separately, the US NIH has published the “NIH Security Best Practices for Controlled-Access Data Subject to the NIH Genomic Data Sharing (GDS) Policy”^5^ (hereafter, “NIH guidelines”), to define the rules for research investigators seeking to access the human genomic and phenotypic data that are maintained in NIH data repositories. It also provides rules for the operation of databases/servers operating in academic organizations or cloud services.

The need for guidelines covering the operation of medical information systems has also been discussed in Japanese government agencies, and two relevant guideline documents have been published. These are the “Security Guidelines for Medical Information Systems” produced by the Ministry of Health, Labour and Welfare^6^ (hereafter, “MHLW guidelines”) and the “Guidelines for Safety Management of Medical Information by Providers of Information Systems and Services Handling Medical Information” jointly produced by the Ministry of Internal Affairs and Communications and the Ministry of Economy, Trade and Industry^7^ (hereafter, “MIC/METI guidelines”. The former guidelines focus primarily on the requirements for administrators in medical institutions or IT service providers, while the latter focus primarily on the requirements for IT service providers, including cloud service providers.

Furthermore, the National Institute of Informatics (NII) has developed a cloud service checklist that is designed to help IT administrators or researchers in academic organizations select the most suitable cloud services. This checklist, which consists of items that we believe IT administrators or researchers in academic organizations should consider when they adopt cloud services, comprehensively covers the issues related to a variety of cloud services, including security, functionality, performance, and law. However, it is sometimes difficult to identify the checklist items that satisfy genome medical science community guidelines, and some guideline requirements are not included in the extant checklist. This issue provided our motivation for creating a cloud service checklist that is customized for genome medical research communities.

In this paper, we therefore provide a cloud service checklist designed to help IT administrators or researchers in academic organizations select the most suitable cloud services, and we have customized this checklist for genome medical research. The resulting customized checklist is designed to help researchers easily find information about the cloud services that satisfy guidelines in genome medical science communities. Additionally, we explore whether many cloud service providers satisfy the requirements or checklist items in the cloud service checklist for genome medical research by analyzing their survey responses.

## Materials and Methods

### GakuNin Cloud Adoption Support Service

**Fig. 1:**
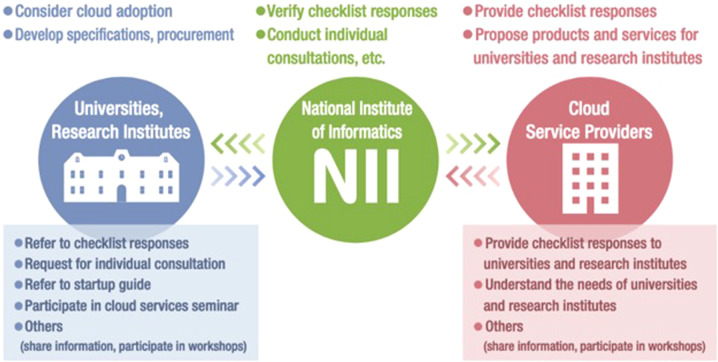
GakuNin Cloud Adoption Support service. GakuNin Cloud Adoption Support Service provides information on cloud adoption and utilization to universities and research institutions.

In Japan, the NII offers “GakuNin Cloud” services to support actual cloud adoption and use in Japanese universities and research institutes. Within GakuNin Cloud services, the GakuNin Cloud Adoption Support Service (CAS) collects, disseminates, and shares the standard processes and information required when universities and research institutes adopt and use cloud services. This service is supported by the NII’s GakuNin Cloud Service Checklist (GCC), which summarizes the relevant issues (reliability, security, contract conditions, etc.) when universities and research institutes adopt cloud services. The NII also collects responses to the GCC from cloud service providers. The responses are verified by the NII and made available to universities and research institutes considering the adoption of such services. Figure 1 depicts an overview of the CAS.

### Checklist organization

The first version of the GCC was formulated by the NII in 2015, and the latest version (V5.1) was published in 2021. An English language version, V5.1E, is available at https://nii-gakunin-cloud.github.io/#checklist. As shown in Table 1, which provides an overview of GCC V5.1, the GCC consists of 112 detailed check items, separated into 19 categories.

**Table 1:** Overview of the GakuNin Cloud Checklist, V5.1.

	Check item	#Detailed check items	Overview
A	Outline of product/service	4	Name of proposed product/service, Product/Service outline
B	Operation results	2	Number of subscribing parties, Service start date
C	Contract application	8	Payment, Licensing system
D	Authentication	3	Authentication with SAML (Shibboleth support), multi-factor authentication
E	Reliability	4	Record of service availability, Planned service outages
F	Support	5	Support desk, Notification for important information
G	Network and communications	9	SINET connection, Communications security
H	Administrative functions	12	Operation status list display, Usage statistics
I	Software environment	4	Supported operating systems, Examples of applications, Operating platforms
J	Scalability	5	Maximum number of servers, Adding resources
K	Data center	7	Physical security, Data center locations
L	Security	11	Incident response, Security measures
M	Data management	9	Data redundancy, Logs
N	Backups	6	Backup services, Restoring from backups
O	Cloud provider reliability	6	Privacy policy, Outsourcing to third parties
P	Contract conditions	6	Clarification of the scope of responsibility, Governing law
Q	Data handling	3	Data intellectual property rights/right to use data, Data deletion
R	Resource continuity	4	Data migration support at the termination of a contract, Server image migration
S	Third-party certification	4	Business continuity, Security

Some specific examples of GCC checklist items are as follows:

- Data center
  To verify the reliability and safety of cloud services, it is first necessary to investigate the data center facility where the cloud services are hosted. Issues of interest here include measures for security, disaster prevention, failure, and disaster response. Additionally, when considering the processing and storage of sensitive data, such as personal and confidential information, it is necessary to confirm the location (country or region) of the data center. Furthermore, since some cloud service providers allow users to choose from among their available data center locations, this function is also important when selecting cloud services. The GCC includes detailed check items that can help resolve such issues. These include, for example, “Are the regions or zones of data centers disclosed? Are data centers located in Japan?” (K6: Data center locations) and “Is it possible for users to specify which data center in which region or zone is used?” (K7: Specification of locations and zones). Here, “K6” and “K7” denote item numbers in the GCC.
- Data management
  Monitoring system statuses or logs is an important issue in the operation of computer systems. Although system administrators manage on-premise system logs, the logs related to cloud services are managed by cloud service providers and may not be accessible to cloud service customers. Thus, when selecting a cloud service, it is necessary to confirm what logs are available for cloud service customers. The GCC includes detailed check items to clarify this issue. These include, for example, “Is a university/research institute permitted the right to use application logs (SaaS), server system logs/operation logs/access logs (IaaS) managed by the provider?” (M2).
- Contract conditions
  Legal issues are important considerations when confirming a contract between a university and a cloud service provider, particularly since many popular cloud services are provided by foreign providers from data centers outside of Japan. As a result, it is necessary to confirm the contract terms and conditions of a contract, including the governing law and court of competent jurisdiction, in case of disputes. To the best of our knowledge, most Japanese universities require their contracts to be governed by the laws of Japan. Accordingly, the GCC includes detailed check items related to legal issues. These include, for example, “Is the governing law for any litigation that may arise taken to be Japanese law?” (P4) and “Is there a court of competent jurisdiction?” (P5).
- Data encryption
  To assure data confidentiality, the data encryption functionality of cloud services should be confirmed. Accordingly, the GCC includes check items related to encryption functions both during network communications (data in flight) and for data stored in cloud storage services (data at rest). These include, for example, “Is guaranteed security of communications between terminal and resources offered?” (G2) and “Is it possible to encrypt stored user data?” (M4).
- Data deletion
  After a cloud user explicitly requests the deletion of data or terminates its cloud contract, the user’s data that are stored in the cloud service infrastructure should not be available to anyone. Accordingly, the GCC includes check items that cover the complete deletion of stored data as well as the applicable deletion methods and the availability of provider-issued deletion certificates. These include, for example, “Is there a guarantee to ensure that no data that a user has clearly deleted, as well as no user information or data belonging to the user remaining after a user has decided to terminate the agreement, are reused? (e.g., all data are deleted in accordance with NIST-SP-800-88) If a data deletion certification can be issued, state this.” (Q2).

### Use-case-oriented checklists

The GCC consists of 112 detailed check items and comprehensively covers issues for a variety of cloud services. However, its comprehensive nature sometimes makes it difficult for users to investigate cloud services by focusing on specific-use cases. Accordingly, we also developed checklists and related documents targeting specific-use cases. For example, the cloud procurement checklist categorizes GCC check items based on working processes (design, specifications, management approval, etc.). A number of specific use-case-oriented checklists are shown below.

- **Checklist for Business Continuity Planning (BCP)/Disaster Recovery (DR)**
  In the abovementioned MEXT survey, more than 70% of queried universities stated that cloud services contribute to their improved business continuity planning (BCP) and disaster recovery (DR)^1^. To support university administrators working to utilize cloud services for BCP/DR, we developed a BCP/DR use-case-oriented checklist. To accomplish this, we first defined three cloud-based system deployment models: an all-in-cloud model, where a whole system is deployed on a cloud; an active-standby model, where a whole system is operated on-premise (active) and a backup system is deployed in a cloud (standby); and the data backup model, where a whole system is operated on-premise and the backup data in the on-premise system is stored in a cloud. The latter two are also called hybrid cloud models. This checklist currently consists of 24 detailed check items, and we plan to provide actual BCP/DR design patterns based on the BCP/DR checklist items.
- **Checklist for online meeting services**
  Online (web-based) meeting services are becoming essential tools not only as temporary expedients, such as during the COVID-19 pandemic, but also in support of future postpandemic working styles. In Japan, most universities switched to online lecture formats in 2020; while many resumed in-person classes in 2022, others plan to continue providing online classes^8^. To support such changes in education and research styles, we developed a use-case-oriented checklist for online meeting services. This checklist is also based on GCC and considers actual use cases of education and research in universities. It also contains input from a telework-related study conducted by the US National Security Agency (NSA)^9^ and various university security experts. In total, the checklist consists of 34 checkpoints, 25 of which were selected from the GCC and nine of which were added later based on the online meeting service requirements of various universities.
- **Checklist for security policy**
  A publication entitled “Examples of Information Security Rules and Regulations for Higher Education Facilities” (EISR) was released by the NII to provide regulations related to the management of confidential information in cloud services^10^. Accordingly, the security policy use-case-oriented checklist was created based on GCC and the abovementioned NII publication. The resulting checklist consists of the check items and best practices that should be considered in terms of information security when public cloud services are used in universities. Specifically, it includes 72 detailed check items, 71 of which were selected from the GCC, and one newly added item based on EISR requirements. While the GCC is organized in terms of cloud service features, this use-case-oriented checklist includes items for cloud service customers, including what those customers should do when using a cloud service. Although its primary purpose is to ensure compliance with security policies, it is also intended to help universities consider security issues when selecting and using cloud services. Additionally, it can reduce the efforts required when defining internal guidelines for cloud adoption in universities.
- **Checklist for high-performance computing (HPC) services**
  Since high-performance computing (HPC) services are actively used in many academic communities, the verification of HPC services is an important issue when users run jobs involving sensitive data. Accordingly, we developed a use-case-oriented checklist for HPC services in collaboration with the RIKEN Center for Computational Science (R-CCS)^11^. This checklist includes 100 detailed check items, 89 of which were selected from GCC and 11 of which were added later based on HPC service requirements.

## Results

### Cloud service checklist for genome medical research

Although the GCC is suitable for use when comprehensively surveying cloud services, its use can sometimes be difficult for researchers searching for the cloud service that is most suitable for running a particular application. For example, genome medical science researchers may need to run applications on cloud services with higher security functions to protect sensitive data, such as personal information, to follow the defined guidelines in their research community. However, it is sometimes difficult to identify the checklist items that satisfy those community guidelines, and some guideline requirements are not included in the GCC. This issue provided our motivation for creating a cloud service checklist that is customized for genome medical research communities. The resulting customized checklist was thus designed to help researchers easily find information about the cloud services that satisfy the guidelines in genome medical science communities.

**Fig. 2:**
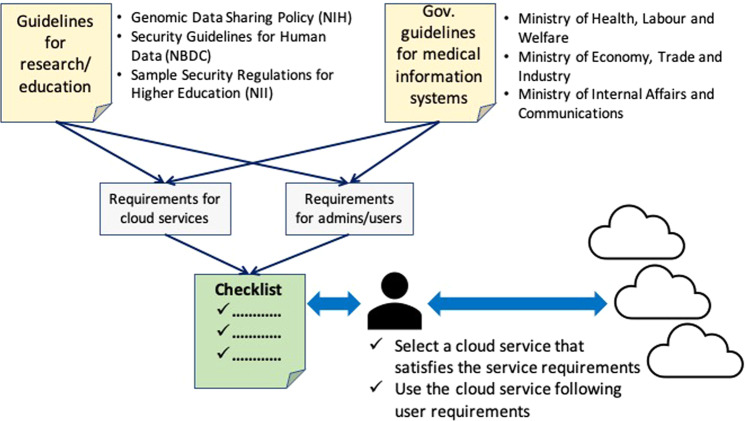
Checklist for genome medical research. Creating the checklist, we surveyed three guidelines used in other academic communities, the NBDC guidelines, the NIH guidelines, and the Examples of Information Security Rules and Regulations for Higher Education Facilities. We also surveyed two government guidelines, the Ministry of Health, Labor and Welfare guidelines and the guidelines from the Ministry of Internal Affairs and Communications and the Ministry of Economy, Trade and Industry.

Figure 2 shows how we created this checklist. First, we surveyed three guidelines used in other academic communities, the NBDC guidelines, the NIH guidelines, and the EISR^10^. The latter document was included, even though it does not focus on genome medical science, because it is used by many universities in Japan when creating security policies in their institutions. We also surveyed two government guidelines^6,7^.

Next, we selected the specific requirements for cloud service providers and customers. In this stage, we assumed that the cloud service customers would be research groups organized by a system administrator and data users, whereby the system administrator provides a suitable application execution environment for data users and the data users analyze data using that application execution environment. Table 2 summarizes the participants in this checklist.

**Table 2:** Genome Medical Research Checklist Participants.

Participants		Definition
Cloud service customer	System administrator	Person(s) in charge of information system management/operation
	Data user	Researchers using genome data
Cloud service provider		Cloud service providers providing cloud services

Finally, we mapped the selected requirements with related items in the GCC by selecting those that discuss specific requirements and then defined the matching requirements for cloud service providers and customers. For example, the NBDC, NIH, and MIC/METI guidelines require an infrastructure (or a cloud service provider) to provide a communication encryption function between cloud servers and customer terminals. Additionally, the MHLW and NBDC guidelines require a user (or a cloud service customer) to access servers via encrypted communication. Based on these inputs, we defined the G2 checklist item in Table 3, which also shows an example of the data deletion checklist Item Q2. Here, it should be noted that since we also found new requirements, which had not been covered in the GCC, new items were added to define those requirements. Table 4 summarizes the items in the checklist, which consists of 46 items, categorized into 13 groups.

**Table 3:** Examples of Checklist Items in the Checklist for Genome Medical Research.

No.	Check item	Cloud service provider	Cloud service customer	Related guideline(s)	Infra.	User
G2	Ensuring secure communications	Provide a function for encrypting communications between the terminal and server. Also, provide details about the encryption method.	Operators: When logging into the server and engaging in outside data communications, all communication routes shall be encrypted using sufficiently secure methods (multiple encryption algorithms listed in the “e-Government Recommended Ciphers List” and secure protocols based on them).	NBDC	X	X
				NIH	X	
				MHLW		X
				MIC/METI	X	
Q2	How to delete data	Clearly state whether there is a guarantee that user-deleted data, user information, and user-owned data will not be retrieved or reused after the user has decided to terminate the contract. Instruct customers on a method for irrecoverably deleting data (including backup data that is no longer needed) in advance. Provide a guarantee that data deleted by customers is irrecoverable and will not be reused. It is desirable to be able to issue a certificate of deletion.	Operators and data users: Before deleting data, ensure that the data in question does not need to be retained for a more extended period based on applicable laws, guidelines, or plans. Data that is no longer needed (e.g., backups, project storage, databases and their backups, archives) must be deleted using a Provider-approved method that makes the data unrecoverable. Also, check the provider’s rules for disposal. Paper and portable storage media that are no longer needed must be disposed of irrecoverably (using a shredder for paper).	NBDC		X
				NIH	X	
				MHLW		X
				MIC/METI	X	X

**Table 4:** Organization of the Checklist for Genome Medical Research.

Check item	Specific check item
Network and communication functions	SINET connection support for SINET connection service, Ensuring secure communications, Access control function, IP address restrictions
Management function	Operation status list display function
Software environment	Operation platform
Data centers	Physical security, Room access management system, Disaster contingency measures, Network failure contingency measures, Data center regions, and Designation of regions and zones
Security	Security policy, Incident responses, Frequency of version upgrades, Provide update information, Automatic security updates, Security measures, Virus definition updates, and Intrusion Detection System (IDS)・Intrusion Prevention System (IPS)
Data management	Right to use (e.g., view) logs, Data encryption, How to manage encryption keys, Redundancy methods, Data access restrictions, Local copy retention, and Data synchronization
Backups	Backup data security
Reliability of cloud provider	Audits by users, Disclosure of service audit results
Terms and conditions	Clarifying the scope of responsibility, Governing law, Court of competent jurisdiction
Data handling	How to delete data
Data transfer	Data migration support at the end of a contract, Securing data at the end of service use
Third-party certification	Security
Misc.	Handling removable information media, Formulation of access control rules, Personnel security management measures for employees, Identification and authentication of users, Peep prevention measures, Physical disposal of storage devices and other equipment for maintenance purposes, Formulation of BCPs

## Discussion

This section explores whether many cloud service providers satisfy the requirements or checklist items in the use-case-oriented checklist for genome medical research (GCR) presented in the previous section. The NII distributes the GCC to cloud service providers and collects the responses. We analyzed responses to our survey and summarized the satisfaction rate relevant to the GCR. Here, the satisfaction rate refers to the percentage of cloud service providers who responded positively to the detailed check items, and our survey consisted of two question types: requests for alternative (Yes/No) answers and descriptive answers.

**Fig. 3:**
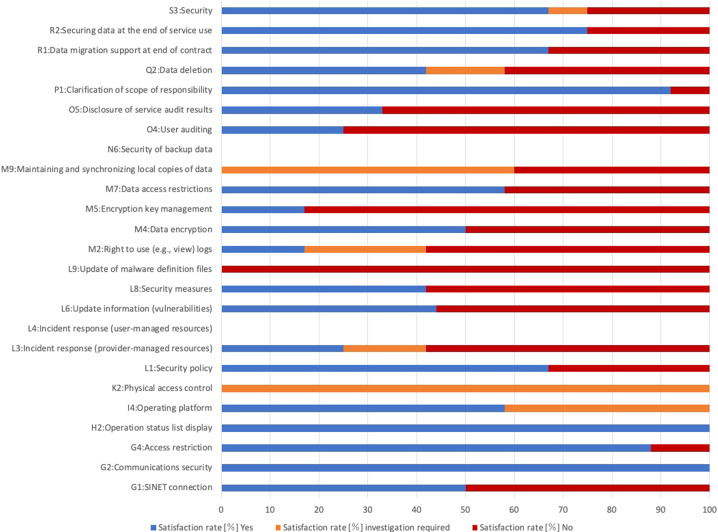
Satisfaction rate for genome medical research checklist. The blue bar denotes the satisfaction rate, which is the percentage of cloud service providers that responded positively to a detailed check item, while the red bar denotes the percentage of cloud service providers that did not. The orange bar indicates the percentage of cloud service providers whose satisfaction rate could not be determined based on the answers we received.

We consider a cloud service provider to be positive in regard to an item if the answer is “Yes” for the alternative question or if the measures related to the question have been sufficiently described in a descriptive answer. Figure 3 summarizes the satisfaction rate for the checklist. The blue bar denotes the satisfaction rate, which is the percentage of cloud service providers that responded positively to a detailed check item (Satisfaction rate [%] Yes), while the red bar denotes the percentage of cloud service providers that did not (Satisfaction rate [%] No). The orange bar indicates the percentage of cloud service providers whose satisfaction rate could not be determined based on the answers we received (Satisfaction rate [%] investigation required).

The results in Fig. 3 show that the satisfaction rate for “G2: Communications security” is 100%. In other words, all the cloud service providers offer sufficient methods for enabling secure communications, such as SSH or SSL/TLS for encryption, AES for file sharing, and SINET L2VPN, Internet Protocol Secure (IPsec), and Secure Sockets Layer Virtual Private Network (SSL-VPN). Notably, SINET L2VPN is a secure and high-speed VPN service offered by the Science Information NETwork (SINET) for Japanese academic communities^12^. Since secure communications are indispensable to access cloud services on the internet, it is essential that cloud service providers offer secure communication methods as a standard service.

On the other hand, we note that for some items, cloud service providers did not disclose the requested information for security reasons. For example, the satisfaction rate for the item “K2: Physical access control”, which asks how physical access to the data center is managed (e.g., IC card authentication, biometric authentication, or physical identification by a security guard), was 0%. In other words, none of the cloud service providers were willing to disclose that information in response to our survey. However, we have also confirmed that they disclose such information, possibly via a nondisclosure agreement with customers, including those who are considering the use of their cloud services.

In addition, the results show that the satisfaction rate for the item “M5: Encryption key management”, which concerns the key management method used for data encryption, was 17%. Here, we note that many cloud service providers use internally managed encryption keys. Hence, such cloud service providers may be unwilling to disclose such information due to security reasons. However, other cloud service providers enable their customers to manage their own data encryption keys, and thus those cases likely explain how the satisfaction rate of 17% could be achieved.

The satisfaction rate for the item “L9: Update of malware definition files”, which asks how frequently definition files are updated if virus or malware detection and protection services are provided, was also 0%. This indicates that none of the cloud service providers were willing to disclose the frequency at which their virus definition files are updated. These cloud service providers therefore deem their customers responsible for updating malware definition files. Additionally, malware detection features are usually provided by third parties, whereby cloud service providers cannot be fully responsible for updating malware definition files. Thus, in such cases, it is possible to minimize related problems on the customer’s side by implementing third-party virus or malware detection and protection software.

## Data Availability

The cloud service checklists discussed in this paper are available at: https://nii-gakunin-cloud.github.io/#checklist.
